# Bayesian and Frequentist Analytical Approaches Using Log-Normal and Gamma Frailty Parametric Models for Breast Cancer Mortality

**DOI:** 10.1155/2020/9076567

**Published:** 2020-02-08

**Authors:** Refah Mohammed Alotaibi, Chris Guure

**Affiliations:** ^1^Mathematical Sciences Department, College of Science, Princess Nourah bint Abdulrahman University, Riyadh, Saudi Arabia; ^2^Department of Biostatistics, School of Public Health, University of Ghana, Legon, Accra, Ghana

## Abstract

One of the major causes of death among females in Saudi Arabia is breast cancer. Newly diagnosed cases of breast cancer among the female population in Saudi Arabia is 19.5%. With this high incidence, it is crucial that we explore the determinants associated with breast cancer among the Saudi Arabia populace—the focus of this current study. The total sample size for this study is 8312 (8172 females and about 140 representing 1.68% males) patients that were diagnosed with advanced breast cancer. These are facility-based cross-sectional data collected over a 9-year period (2004 to 2013) from a routine health information system database. The data were obtained from the Saudi Cancer Registry (SCR). Both descriptive and inferential (Cox with log-normal and gamma frailties) statistics were conducted. The deviance information criterion (DIC), Watanabe–Akaike information criterion (WAIC), Bayesian information criterion (BIC), and Akaike information criterion were used to evaluate or discriminate between models. For all the six models fitted, the models which combined the fixed and random effects performed better than those with only the fixed effects. This is so because those models had smaller AIC and BIC values. The analyses were done using R and the INLA statistical software. There are evident disparities by regions with Riyadh, Makkah, and Eastern Province having the highest number of cancer patients at 28%, 26%, and 20% respectively. Grade II (46%) and Grade III (45%) are the most common cancer grades. Left paired site laterality (51%) and regional extent (52%) were also most common characteristics. Overall marital status, grade, and cancer extent increased the risk of a cancer patient dying. Those that were married had a hazard ratio of 1.36 (95% CI: 1.03–1.80) while widowed had a hazard ratio of 1.57 (95% CI: 1.14–2.18). Both the married and widowed were at higher risk of dying with cancer relative to respondents who had divorced. For grade, the risk was higher for all the levels, that is, Grade I (Well diff) (HR = 7.11, 95% CI: 3.32–15.23), Grade II (Mod diff) (HR = 7.89, 95% CI: 3.88–16.06), Grade III (Poor diff) (HR = 5.90, 95% CI (2.91–11.96), and Grade IV (Undiff) (HR = 5.44, 95% (2.48–11.9), relative to B-cell. These findings provide empirical evidence that information about individual patients and their region of residence is an important contributor in understanding the inequalities in cancer mortalities and that the application of robust statistical methodologies is also needed to better understand these issues well.

## 1. Introduction

Cancer of the breast known as breast cancer occurs when cells that are in the breast grow out of control, usually forming a tumour that can be detected by X-ray or felt as a lump. Malignant tumours can metastasize to invade surrounding tissues or spread to distant areas of the body, including the blood and lymph system. Several studies have identified breast cancer as one of the causes of death that occur in the Western world [1]. It is also observed as one of the common malignancies among the people of Saudi Arabia, where it affects 21.8% of women. The Saudi Cancer Registry at the King Faisal Specialist Hospital and Research Centre stipulates that about 930 cancer cases are diagnosed yearly in Saudi Arabia or around 19.5% of Saudi and non-Saudi women.

Al-Qahtani [2] found breast cancer to be among the top two common malignancies among Saudi Arabia women. In 2010, it was estimated to be the 9^th^ most common cause of women mortality in the Kingdom of Saudi. About 27.4% (1,473 out of 5,378) newly diagnosed cancers were female breast cancers [3, 4]. Ibrahim et al. [5] predicted that in the next decade, there will be an increase due to population growth and aging.

The 2012 report (annual) of the Saudi National Cancer Registry recorded about 26.4% of women less than 40 years compared to 6.5% the United States of America. The diagnoses of breast cancer before age 40 are linked to poorer prognoses, more aggressive cancers, and higher mortality and recurrence rates than diagnoses after age 40 [6, 7]. Anders et al. [8] found that seven percent of female breast cancer are diagnosed before they reach the age of 40. Also, survival rates among the younger women are much worse than that for the elderly. They also found that the incidence of breast cancer among the male population was estimated at 1/100^th^ of the rate for females.

Few studies have investigated factors that influence cancer progression among women in the Saudi population. Those that have done so have used mostly standard survival models such as Cox without considering dependencies of the data collected neither do they account for variations that may exist among participants with either similar or different characteristics. Though this approach may be viewed as being complex, it is necessary to help in determining significant factors of breast cancer.

## 2. Methods

### 2.1. Data Source and Variables

We used data from the Saudi Cancer Registry (SCR) to conduct our study. The Saudi Cancer Registry (SCR) is responsible for cancer data collected across the cancer registries from all the administrative regions in the Kingdom. These regions include Riyadh, the Eastern and Northern regions, Makkah, Madinah, Qassim, Hail, Jouf, Tabouk, Najran, Baha, Asir, and Jezan. These regions contain information of the people in the country. The data obtained and used in this analysis are from all the hospitals under the Ministry of Health and also from the private hospitals, clinics, and laboratories. The data are abstracted from patients who are categorised as cancer patients via clinical, histopathological, and radiological diagnoses. The SCR supervises all its offices across the regions to ensure accuracy and quality controls for data collection.

The current data contain information on 8,312 patients (8,172 women and 140 men). These patients were diagnosed with advanced breast cancer between the years 2004 and 2013. Data collected include patients' survival time, censorship, sex of participant, age of the participant, marital status of the participant, address of the participant, nationality of the participant, and tumour details of the participant, such as laterality (primary site), behaviour, grade, stage (extent), and topography of the participant. The primary site (topography) and histology (morphology) of the malignancies were identified using the International Classification of Diseases for Oncology 3rd Edition (World Health Organization, 2000).

Data entry was carried out using CanReg 4 (IACR) software. After removing 2,880 patients due to missing information, the remaining 5,432 patients were analyzed. We conducted a serious of steps, including verifying sites, morphologies, and staging, to ensure data quality and accuracy for all regions. We also verified case linkage (tumour and patient) and performed data consolidation. Other data collected included personal identification, demographic information, and tumour details.

The outcome variable was defined as survival time in years for all the patients who were diagnosed with breast cancer. Those who died as a result of breast cancer were the patients classified as having had the event and assigned the number 1. Patients who either dropped out of the study, did not die within the study period, or died from other diseases unrelated to breast cancer were censored (using right censoring mechanism as illustrated in equation (8)) and assigned the number 0.

### 2.2. Statistical Approach

Series of Weibull models were fitted, using both Bayesian and frequentist frameworks. The standard Weibull model was fitted first and denoted as Model 1. Random terms were added to capture variation at the regional level (Model 2), and extent was captured as Model 3. The decision to use the Weibull regression model was premise on the bases of previous knowledge on the distribution of breast cancer deaths [9]. The Weibull model [10] is a well-established model appropriate for modelling hazards that are either monotonically decreasing or increasing [11]. Application of parametric survival models to model breast cancer has been carried out [9, 12] and elaborated on the advantages of using this model to account for censoring in data from administrative and historical databases [11]. Based on the model formulated by Nasejje et al. [13], which was parameterized from Martino et al. [14], we utilized the proportional hazards model of the form: *h*_*i*_(*t*)=*h*_0_(*t*)exp(*η*_*i*_), *t* > 0, where the baseline hazard is represented by *h*_0_(·) and the predictors by *η*_*i*_. In this analysis, we assume that the data follow the right censoring mechanism, where individuals who die as a result of breast cancer are said to have had the event and all others (death not related to breast cancer, dropouts, and lost to follow-up) are said to have censored (have not had the event of interest). If we specify a Weibull model for the baseline hazard, then we have *h*_0_(*t*)=*λt*^*λ*−1^, *λ* > 0, while log likelihood for the observation (*t*_*i*_, *δ*_*i*_) taking into consideration the right censored approach can be further formulated as

(1) $$\begin{array}{c} l=\delta_{i}\log\;h\left(t_{i}\right)-\int_{0}^{t_{i}}h\left(u\right)\text{d}u, \end{array}$$

(2) $$\begin{array}{c} l=\delta_{i}\log\left(\lambda_{i}+\left(\lambda -1\right)\log\;t_{i}+\eta_{i}-\exp\left(\eta_{i}\right)t_{i}^{\alpha }\right.. \end{array}$$

Following the definition of Martino et al. [14], we let the predictors to be represented by *η*_*i*_, which assumes an additive structured form. We extend equation (2) to the Weibull regression model. Expressing the latent field *x*, in terms of the predictor *η*_*i*_, the standard Weibull regression is expressed as

(3) $$\begin{array}{c} h_{i}=\lambda t^{\lambda -1}\exp\left(\eta_{i}\right),\\ \eta_{i}=\beta_{0}+\overset{n=9}{\underset{i=1}{\sum }}\beta_{i}x_{j},\;i=1,\ldots ,9, \end{array}$$

where *β*_0_ ~ *N*(0, 0.01^−1^) and *β*={*β*Sex, *β*_2_Lat, *β*_3_Top, *β*_4_Marita, *β*_5_Grade, *β*_6_Ext, *β*_7_Cause, *β*_8_AddressCode}=*N*(0, *I*).

We specify a Gaussian distribution with hyperparameters *θ*=(*θ*_1_, *θ*_2_) as priors for the Weibull regression coefficients and the gamma distribution Γ(*a*, *b*), with mean = *a*/*b* and variance = *a*/*b*^2^ as priors for *λ*. Flat priors were also assigned to *τ*=Γ(1,1).

### 2.3. Conditional Survival Models (Gamma and Log-Normal Frailty Models)

Implementing standard survival methodologies without accounting for the correlational structure has methodological and practical implications. These approaches methodologically ignore the clustering effect among communities within the same region which has the potential to bias the estimation due to violations of the statistical assumptions of independence. It is for this reason that conditional (frailty) models which allow for the correlational survival experiences of patients and therefore provide better estimates of the coefficients and their standard errors were developed. Broadly, the importance of frailty models cannot be overemphasized as they are used to estimate the effect of unmeasured factors on the risk of cancer deaths. The frailty term in survival models is considered a random variable over/of the population and constitutes a frailty distribution.

In this work, we extend marginal survival models that have been extensively applied in modelling breast cancer risk factors elsewhere [15, 16] by including the random effect (the frailty) which is acting multiplicatively on the hazards function, as demonstrated elsewhere [14, 17–20].

In conditional survival models, it is argued that individual risk of death is a function of the measured factors and that of a random perturbation on the baseline hazard as a result of the unobserved effects. In this work, the standard Weibull function was extended to include the frailty term, which was constructed as described below.

Let *t*_*ij*_ be the survival time for the *j*^th^ cancer patient in the *i*^th^ region. In this work, say patients' *j*, where *j*=1,…, *n*, are nested within regions (address code), that is, *i*=1,…, *s*. We further assumed that these *t*_*ij*_ follow the Weibull regression model. The Weibull model can mathematically be expressed as

(4) $$\begin{array}{c} t_{ij}=\text{Weibull}\left(\lambda ,\;\eta_{ij}\right). \end{array}$$

We conditioned the hazard function of *t*_*ij*_ on the parameter *η*_*ij*_ and the Weibull scale parameter *λ*. This is mathematically expressed as

(5) $$\begin{array}{c} h\left(\left.t_{ij}\right|\eta_{ij},\lambda \right)=\;\lambda t^{\lambda -1}\exp\left[\eta_{ij}\right], \end{array}$$

where *η*_*ij*_= *β*_0_+ *Z*_*ij*_^*T*^*β* in which *β* is a *p* × 1 vector of regression coefficients, *β*_0_ is the intercept, and *z*_*ij*_ is a *p* × 1 covariate vector.

A frailty model that is expressed in a univariate case introduces what is referred to as an unobserved multiplicative effect on the hazard [21] such that it can be conditioned on the frailty as

(6) $$\begin{array}{c} h\left(\left.t_{ij}\right|\eta_{ij},\lambda ,\;\psi_{i}\right)=\;\lambda t^{\lambda -1}\exp\left[\eta_{ij}\right]\psi_{i}, \end{array}$$

where *ψ*_*i*_ (for the *i*^th^ group) is taking to be a random positive quantity which has a mean of 1 and variance say, *θ*. Individuals with the frailty parameter of *ψ*_*i*_ > 1 are said to be frail and with an increased risk of failure (breast cancer mortality) due to unexplained reasons by the covariates while individuals with *ψ*_*i*_ < 1 are said to be less frail and with a decreased risk of failure (tern to leave longer). Due to the multiplicative nature of *ψ*_*i*_, one can think of a frailty as representing the cumulative effect of one or more omitted covariates as illustrated in equation (6) (for details on frailty modelling, refer to [22–27] and [28]).

Using the Laplace approximation modelling technique as detailed in Martino et al. [14] and Martino and Rue [29], we formulate the Weibull regression with the frailty parameter as

(7) $$\begin{array}{c} \eta_{ij}=\beta_{0}+\beta \text{Sex}+\beta_{2}\text{Lat}+\beta_{3}\text{Top}+\beta_{4}\text{Marita}+\beta_{5}\text{Grade}+\beta_{6}\text{Ext}+\beta_{7}\text{Cause}+\psi_{i}, \end{array}$$

where *ψ*_*i*_=log(*μ*_*i*_) ~ *N*(0, *t*^−1^) with completed likelihood assuming a right censoring mechanism given by

(8) $$\begin{array}{c} L\left(\left.\beta ,\lambda \right|D\right)=\overset{n}{\underset{i=1}{\prod }}\overset{s}{\underset{j=1}{\prod }}\left(\lambda t^{\lambda -1}\exp\;{\left(\eta_{ij}\right)}^{\delta_{ij}}\exp\left(-\exp\left(\eta_{ij}\right)t_{ij}^{\lambda }\right)\right). \end{array}$$

Log-normal frailty was used to establish the association that exists between survival times that maybe related to patients from the same region. Normal priors were assigned to the regression coefficients (betas) while flat priors were assigned to *τ* and *λ* parameters.

We allowed *δ*_*ij*_ to represent censoring with a value 1 if the *j*^th^ patient in the *i*^th^ region dies and 0 otherwise with *D*=(*t*, *x*, *δ*, *ψ*).

However, fitting gamma frailty models in INLA, which is based on the Gaussian Markov field, requires employing a different technique. Thus, we reparametrized the Weibull function as advised by Martins and Rue [30]. Parameterization of the Weibull survival model to include gamma frailty was achieved by imposing restrictions on the non-Gaussian components of the latent field so that this distribution could be very well approximated by a Gaussian density. This correction is appropriate, especially in light of adding flexibility around a Gaussian distribution, as advised by Martins and Rue [30].

Using equation (7), with *η*_*ij*_ representing the linear predictors as described in Martins and Rue [30], the gamma frailty term *ψ*_*i*_ was further reconstructed as log(*CT*_*i*_)=*k*(*ψ*_*i*_ − weibul(*ψ*_*i*_)+(*τ*_*ψ*_(*k*)/2)*ψ*_*i*_ − *μ*_*ψ*_(*k*))^2^+const, where *μ*_*ψ*_(*k*) is the mean and *τ*_*ψ*_(*k*) is the precision parameter of the Gaussian approximation to the log gamma random term.

### 2.4. Bayesian Analysis Using a Deterministic Approach (INLA)

The Bayesian approach is implemented using integrated nested Laplace approximation approach (INLA). INLA provides approximations of simulation-free Bayesian inference using integrated nested Laplace approximations. The technique has found favour in modelling works that involve complex models, as it has been known to circumvent computational cost which is a big challenge in most sampling-based modelling techniques [14, 30, 31]. Modelling in INLA is based on directly approximating the posterior marginal; *π*(*x*_*i*_/*y*) and *π*(*θ*_*i*_/*y*) from the posterior distribution;

(9) $$\begin{array}{c} \pi \left(x,\frac{\theta }{y}\right)\propto \pi \left(\frac{y}{x}\right)\pi \left(\frac{x}{\theta }\right)\pi \left(\theta \right). \end{array}$$

The approximated posterior marginals are used for computing summary statistics of interest, including the posterior means, variances, or quantiles. The strategy involves three components: the data, the latent model, and the hyperparameter. The data in INLA are generated from the original components of the observation *x* and hyperparameter *θ*. This is also part of the likelihood function defined as *π*(*y*/*x*, *θ*).

INLA is principally based on statistical inference for latent Gaussian Markov random field (GMRF) models. GMRF models are hierarchical models involving several steps [32–34]. The first steps include finding a distributional assumption for the observed *y*, which is usually conditioned on some latent parameters *η* and some parameters *θ* formulated as

(10) $$\begin{array}{c} \pi \left(\frac{y}{\eta_{i}},\theta \right)=\Pi_{j}\pi \left(\frac{y_{j}}{\eta_{j}},\theta_{1}\right). \end{array}$$

The latent model component is the most appealing part of the INLA framework, especially as it relates to how frailty modelling works. The latent modelling component is drawn from the unobserved components *x*. The unobserved component also represents the structured random effect (region effects) and the unstructured random effects representing effects at individual and group levels estimated together with predictors. The latent components are linked to the likelihood through linear predictors, as shown in equation (12) according to [30].

(11) $$\begin{array}{c} \frac{y_{i}}{\eta_{i}},\theta_{1}∼\pi \left(\frac{y_{j}}{\eta_{j}},\theta_{1}\right), \end{array}$$

(12) $$\begin{array}{c} \eta_{j}=\text{offset}+\overset{n_{f}-1}{\underset{k=0}{\sum }}w_{ki}f_{k}\left(c_{ki}\right)+z_{i}^{T}\beta +\varepsilon_{i},\;i=0,\ldots ,n_{\eta }-1,j=J. \end{array}$$

The third component is on the hyperparameters (*θ*=*θ*_1_, *θ*_2_), which are very important components/elements/constituents of the Bayesian framework. The hyperparameters are helpful in specifying the prior distribution and are important for determining precision for unobserved factors.

### 2.5. Description of Analysis

Descriptive statistics such as means and standard deviations were used to analyze measures such as age of the patient. Frequencies as well as proportions were used to describe categorical variables. The Kaplan–Meier survival curve and the log-rank test were carried out to determine differences in survival by gender, extent, and address code. All variables that were included in the final analysis for each of the models were observed to be either significant at the simple regression modelling stage or were found to be a significant predictor of our outcome from previous findings (literature).

From the Bayesian perspective, associated factors of breast cancer mortality were modelled using the integrated nested Laplace approximation (INLA) approach via a shared gamma and log-normal frailty models. Estimates generated from INLA were exponentiated to obtain posterior Bayesian summaries which were reported as hazard ratios with their associated 95% credible intervals. This approach enabled us to account for the unmeasured factors and further allowed us to compare the variances between patients, regions, and tumor extent. A frailty variance that is greater than one suggests a higher rate for the event (or shorter survival times) than would be predicted under the basic Weibull model, while that less than one suggests a lower rate (longer survival times). A 95% credible interval that excludes one is deemed to be significant. Comparison of models and their fit was assessed via the deviance information criterion (DIC) and Watanabe–Akaike information criterion (WAIC) in the Bayesian paradigm. Models based on the frequentist approach were compared using the Akaike information criterion and the Bayesian information criterion. For the Bayesian approach, marginal log likelihoods were extracted and used to compute the AIC outside the INLA environment. Using the Computed DIC from the Bayesian approach and the extracted AIC from a frequentist approach, we were able to determine the best fit model between the Bayesian and frequentist fitted models. All statistical analyses were performed following the R (frailtypack) package, Rondeau et al. [35] for the frequentist approach, while the Bayesian approach was implemented using a deterministic based approach, known as INLA, a variant of R open source software. Before running the models, we scaled the survival time to the maximum of 1 (one) similar to variable centring (time/time max). This was done in order for the INLA software to converge or else it crashes. We used the Bayesian statistical approach in order to obtain posterior estimates which are easy and straightforwardly interpretable mainly because of the random nature of the parameters in the Bayesian as opposed to the frequentist framework.

## 3. Results

In Table 1, we provide a summary of the covariates included in this analysis. There are evident disparities by regions (address code), with Riyadh, Makkah, and Eastern Province having the highest number of cancer patients at 28.39%, 26.30%, and 20.47%, respectively. Grade II (5.86%) and Grade III (4.63%) are the most common cancer grades. Left paired site laterality (50.92%) and regional extent (52.32%) were also most common characteristics. In general, there were 13.19% cancer deaths and 86.81% noncancer deaths. The majority of the participants were females (98.73%) with 1.27% being males. Quite a number of the participants were divorced (84.28%) with only 2.51% being married.

### 3.1. Model Assessment and Evaluation

Table 2 provides model selection values obtained for both the marginal and conditional survival models with the covariates but with different frailty distributions. For all the six models fitted, the models which combined fixed and random effects performed better than those with only the fixed effects because these models had smaller DIC and BIC values, which shows the importance of both the combined fixed and random effects approach in explaining cancer patient survival. For the frequentist and Bayesian approaches, the best model was M2a and M2b, when log-normal frailty terms were specified for both. To be consistent and to compare the two approaches on the same scale, we computed the Bayesian information criterion for all the Bayesian models. Thus, we used the BIC as opposed to the DIC to discriminate models across the two approaches. Using the BIC values, model M2b had the smallest BIC value of 13913.35, compared to the standard fixed effect Weibull model M1b (15057.52) and gamma distributed frailty Weibull model (BIC = 218424.1). Moving to the frequentist models, both BIC and AIC were used to evaluate models. Using the BIC values, the model that combined fixed and log-normal random effects (M2a; BIC = 2615.16) were better compared to the fixed effect (BIC = 2661.71) and gamma distributed random effect model (BIC = 2673.29). In both approaches, the gamma frailty model did not fit the data well as observed using AIC/BIC and DIC. Thus, interpretation of the results was based on M1a, M2a, and M2b (see Table 2). We feel it is the parametrization via the INLA software that might have inflated the BIC and DIC values under the Bayesian approach. Due to the large values obtained for the BIC and DIC via the Bayesian fitted models, we anticipate model fitting issues, and this also reveals lack of data support especially for the proposed gamma frailty distributed model.

### 3.2. Fixed Effect Model (Standard Weibull Model Results)

We estimated two models that assume that cancer patients from same region have uncorrelated survival risk. Furthermore, two additional models were constructed to control for frailty effect at the regional level. This strategy enabled us to examine the effects of unobserved factor estimates of the known determinants for cancer.

Presented in Table 3 are the fixed effects results from the two standard Weibull models constructed to analyze cancer patients' data. Overall marital status, grade, cause of death, and cancer extent increased the risk of a cancer patient dying. Respondents who are married had a hazard ratio (HR) of 1.36 and a 95% CI (1.03–1.80) and that of the widowed had a HR of 1.57 and a 95% CI (1.14–2.18). Both of them were at a higher risk of dying with cancer relative to those respondents who were divorced. For grade, the risk was higher for all the levels i.e Grade I (Well diff) had an HR of 7.11 and a 95% CI (3.32–15.23) Grade II (Mod diff) HR of 7.89 and 95%CI (3.88–16.06), Grade III (Poor diff) an HR of 5.90 95% CI (2.91–11.96), Grade IV (Undiff) an HR of 5.44 and 95% CI (2.48–11.9) were relative to B-cells. While the extent of cancer increased the hazard of death relative to metastasis distance for localized and Regional levels with hazard ratio of 1.73 95% CI (1.45–2.07) and 1.60 and 95% CI (1.43–1.79) respectively. Other factors had a protective effect on cancer patients. For instance, after controlled for other factors, auxiliary tail with an HR of 0.38; 95% CI (0.18–0.80) and breast No's with an HR of 0.73 and a 95% (0.55–0.97) decreased the hazard risk of cancer patient compared to nipple topographical areas. Other topographical factors were found not to be statistically significant. Table 3 provides more information about the determinants included in this analysis.

### 3.3. Comparing Conditional Models

Tables 4 and 5 provide results from the two conditional models fitted to control for unobserved (regional) effects. The estimated variance associated with the frailty effect in the Weibull log-normal frailty model presented in Table 4 was less than 0.01 in Model 2a and 5.2 in Model 2b. For Models 3a and 3b as presented, which included the gamma frailty parameter, the frailty variance was 0.98 for Model 3a and negligible for Model 3b. The frailty parameter values for Model 2b and Model 3a are statistically significant indicating that survival risks among cancer patients vary as a result of unobserved effects which is shared by members of the same region. Following the work of Govindarajulu et al. [22], the estimated frailty as shown in Model 2b indicates that a cancer patient's death in a particular region increases the risk of the death for the index cancer patient by $\left(\text{exp}\left(\sqrt{5.2}\;\right)=9.78\right)$ times relative to the overall risk of breast cancer patient's death. Also, the estimated frailty variance value of 0.98, for Model 3a, denotes that every death is associated with $\left(\text{exp}\left(\sqrt{0.98}\;\right)=2.69\right)$ increased risk of the indexed cancer patient dying relative to the average risk of death. Though the standard errors for the hazard ratios of the covariates are generally higher with the frailty Weibull models as compared to the standard model with higher confidence intervals for grade, for instance, the impact of the frailty term on parameter estimates is small and does not change the significance of any of the parameter estimates in the model. Similarly, the magnitude of the effect of the determinants included as covariates in the model is largely unchanged in the presence of the random term known as frailty.

However, though the two modelling strategies produced similar results, conflicting results were also observed. For instance, marital status and cancer extent exhibited consistently reduced risk of cancer deaths compared to what was reported using frequentist approach. There were also considerable changes in the effects of selected covariates used in the frequentist when analyzed using the Bayesian approach. For example, the effect of age, paired site as a measure of literality, and gender on the risk of death became apparent as the confidence intervals of the two variables did not include 1 (one) in the Bayesian approach. Overall, the magnitude effect of topography and grade became nonsignificant when analyzed using the Bayesian approach. We observed that the measures of effect for topography and literality had an increase effect under the Bayesian approach, while a decreased effect was observed with the frequentist approach.

The effect of frailty models can be demonstrated by looking at the effect of marital status on the risk of death of the cancer patient. Results from the standard model showed that the effect of being widowed reduced from 5.57 to 5.55 in the frailty model. This is a lesser effect reduction of about 2%.

## 4. Discussion and Conclusion

We have examined, in this paper, the effects of demographics, extent, topography, grade, and region on breast cancer patient risk of dying in Saudi Arabia. The study compared two advanced statistical approaches that have been used to correct for dependencies in data structures. As demonstrated in this work, marginal survival models (fixed effects models) can be extended to conditional survival models by introducing a random term in the regression model which is later modelled, either through a semiparametric or a parametric approach [19, 36]. These types of models are used more, particularly in medical studies where the patient's levels are recurrent in nature and have correlated biomedical data. The history of application for these types of models in medical statistical studies include under five studies [19], cancer studies [37, 38], kidney transplants [39], and genetic studies [22].

Our results suggest that there is evidence of some variation among regions in the risk of cancer mortality that may not be accounted for by the measured factors. The magnitude of effects of the covariates is smaller, and the standard errors are slightly inflated with the frailty models compared to the standard models. As noted, the frailty term addition in the model did not change the direction of the effect of the covariates and at best the estimates remained the same throughout the models, and substantive conclusions from the Weibull Standard models remain valid. Consistent with findings from other studies that applied both frequentist [18, 40] and Bayesian approach [36, 41] separately, the presence of heterogeneity in our study acting at the region level, as determined by the two approaches, varied. Gohari et al. [18] in Iran reported the frailty relative risk of 7.2 in patients with breast cancer, while Gorfine et al. [42] established that accounting for heterogeneity improved the ability of the model to predict the risk of developing a disease over time based on carrier status. Bakhshi et al. [40] proved that cure and frailty models were better than the Cox model to estimate patient survival probabilities.

On the other hand, the presence of unobserved effects in this study reflects a different level of factors that can be categorized as genetic or behavioral, occurring at individual and regional levels, which may not have been measured. We are, however, confident that the other factors that were not considered but helped to reduce the unobserved household effect among cancer mortality risk may have details of specific family history, education background, type of treatment, and progesterone receptor (PR) [40, 43] among other factors.

This modelling approach is an extension of the generalized mixed effects model and can be classified as a generalized survival linear mixed model. These types of models have a complex structure which is easily exploited using the frequentist approach and now the Bayesian approach as demonstrated in this work due to the availability of statistical software. Though our approach is complex, the results obtained are consistent with what has been reported elsewhere. Similar to this study, metastasis increased the effects on the hazard of the event in a study conducted in Iran [40].

We have also demonstrated that some of the effects of the region level variables are difficult to understand. For instance, in this study, topography and grade were found not to be statistically significantly associated with cancer mortality. This is in contrast to what is biologically known and reported elsewhere, where lesion [44] and grade [45] were reported to be strong breast cancer risk factors.

However, this study shows that marital status is predictive of breast cancer mortality risks, and it appeared likely to be a surrogate for cluster effect at regional level including other contextual factors that may not have been examined in this study.

## 5. Strength and Limitations

This research has a number of limitations that need to be pointed out. The power of the study would have improved if spatial temporality was incorporated in the analysis strategy to account for possible spatial effect. Conflicting evidence in case of age, paired site as a measure of literality, and gender on the risk of death revealed inherit differences that could be attributed to differences in computational strategies. INLA being a deterministic strategy, though fast, required reparametrization of the likelihood and the prior distribution so that gamma frailty which is a non-Gaussian distributed parameter can be specified and handled appropriately. We feel that it is this parametrization that could have affected the results especially in instances where ambiguous values were obtained. Due to the large values obtained for the BIC and DIC via the Bayesian fitted models, we anticipate model fitting issues, so our estimates should be interpreted with caution. Furthermore, ambiguous and unrealistic estimates obtained in the Bayesian approach revealed lack of data support to the proposed gamma frailty distributed model contrary to what is assumed elsewhere [19, 46]. In this work, the gamma distribution with parameters (kappa, kappa) assumed a fixed expected value of 1 (one) and variance 1/kappa. However, more discrepancy was also noted between log-normal frailty models fitted using the two approaches.

Notwithstanding the above limitations, the findings as presented show a further step toward an improved understanding of the risk of breast cancer survival among the people of Saudi Arabia. This study has provided further evidence for the argument that information about individual patients and region is important in understanding inequalities in cancer mortalities and the application of robust statistical methodologies is also possible. Unlike the Bayesian approach, the frequentist approach via both the gamma and the log-normal frailties did not exhibit any problem with the fit of the data. This shows that the frequentist approach exhibits a good fit to the breast cancer data when modelled through the Weibull proportional regression with frailty.

## Figures and Tables

**Table 1:** Descriptive distribution of the breast cancer patients' characteristics.

Variable	Frequency	%
Marital status		
Married	135	2.51
Divorced	4525	84.28
Single	318	5.92
Widowed	391	7.28

Sex		
Female	5301	98.73
Male	68	1.27

Address code		
Asir	253	4.71
Baha	75	1.40
Eastern	1099	20.47
Hail	99	1.84
International	8	0.15
Jazan	130	2.42
Jouf	69	1.29
Madinah	265	4.94
Makkah	1412	26.30
Najran	47	0.88
Northern	37	0.69
Qassim	236	4.40
Riyadh	1524	28.39
Tabuk	115	4.71

Topography		
C50.0 nipple	128	2.38
C50.1 central portion	194	3.61
c50.2 upper-inner	372	6.93
c50.3 lower inner	195	3.63
c50.4 upper outer	1319	24.57
c50.5 lower-outer	251	4.67
c50.6 axillary tail	46	0.86
c50.8 overl. lesion	1037	19.31
c50.9 Breast, NOS	1827	34.03

Grade		
B-Cell	11	0.20
Grade I (Well diff)	399	7.43
Grade II (Mod diff)	2462	45.86
Grade III (Poor diff)	2396	44.63
Grade IV (Undiff anaplastic)	101	1.88

Laterality		
Bilateral invasive	55	1.02
Left paired site	2734	50.92
Late	13	0.24
Right	2567	47.81

Cause of death		
Cancer	673	12.53
Not cancer	4696	87.47

Extent		
Distant metastasis	911	16.97
Localised	1649	30.71
Regional	2809	52.32

Status		
Dead	708	13.19
Alive	4661	86.81

**Table 2:** Model comparison with DIC and AIC values.

No	Model	BIC	AIC	LLR (df), *p* value	
Frequentist fitted models					

1	M1a: standard Weibull	2661.71	2630.05	2296 (36), *p* < 0.001	
2	M2a: Weibull with log-normal frailty	2615.16	2615.16	2287 (24), *p* < 0.001	
3	M3a: Weibull with gamma frailty	2673.29	2628.17	2296.14 (35), *p* < 0.001	

	Model	BIC	DIC	Marginal likelihood	Effective parameters
Bayesian fitted models					

4	M1b: Bayesian standard Weibull	15057.52	Infinite	−6905.77	145.07
5	M2b: Bayesian Weibull with log-normal frailty	13913.35	13456.85	−6806.38,	35.0
6	M3b: Bayesian Weibull with gamma frailty	218424.1	3597.29	−20118.03	30117.4

**Table 3:** Standard Weibull model (Model 1).

Variables	Standard Weibull model					
	M1a (Frequentist)			M1b (Bayesian approach)		
	HR	2.5%	97.5%	HR	2.5%	97.5%
Sex						
Female	Ref					
Male	0.96	0.64	1.44	1.36	1.03	1.76
Age	0.99	0.99	1.00	1.00	1.00	1.01
Marital status						
Divorce	Ref					
Married	1.36	1.03	1.8	0.79	0.66	0.96
Single	1.08	0.75	1.57	0.79	0.63	0.99
Widowed	1.57	1.14	2.18	0.72	0.59	0.9
Topography						
C50.0 nipple	Ref					
C50.1 central portion of breast	1.00	0.67	1.50	1.14	0.90	1.45
C50.2 upper-inner quadrant of breast	1.05	0.73	1.50	1.16	0.93	1.44
C50.3 lower-inner quadrant of breast	0.95	0.64	1.40	1.23	0.97	1.57
C50.4 upper-outer quadrant of breast	1.01	0.75	1.35	1.12	0.92	1.37
C50.5 lower-outer quadrant of breast	1.01	0.68	1.50	1.02	0.82	1.29
C50.6 axillary tail of breast	0.38	0.18	0.80	1.11	0.77	1.57
C50.8 overl. lesion of breast	0.82	0.61	1.10	1.13	0.92	1.38
C50.9 breast, NOS	0.73	0.55	0.97	1.16	0.96	1.42
Grade						
B-Cell	Ref					
Grade I (Well diff)	7.11	3.32	15.23	1.02	0.59	1.87
Grade II (Mod diff)	7.89	3.88	16.06	1.02	0.59	1.86
Grade III (Poor diff)	5.9	2.91	11.96	1.05	0.61	1.92
Grade IV (Undiff anaplastic)	5.44	2.48	11.9	0.92	0.52	1.74
Laterality						
Bilateral invasive	Ref					
Left	0.92	0.61	1.39	1.06	0.8	1.43
Paired site, late	0.78	0.36	1.73	2.65	1.2	5.35
Right	0.94	0.62	1.42	1.03	0.78	1.39
Extent						
Distant metastasis	Ref					
Localised	1.73	1.45	2.07	0.90	0.82	0.99
Regional	1.60	1.43	1.79	0.88	0.80	0.96

**Table 4:** Weibull with log-normal frailty (Model 2).

Variables	Weibull with log-normal frailty					
	Frequentist			Bayesian approach		
	HR	2.5%	97.5%	HR	2.5%	97.5%
Sex						
Female	Ref					
Male	0.95	0.63	1.43	1.36	1.03	1.76
Age	0.99	0.99	1.00	1.00	1.00	1.01
Marital status						
Divorced	Ref					
Married	1.38	1.05	1.83	0.79	0.66	0.96
Single	1.08	0.75	1.57	0.79	0.63	0.99
Widowed	1.55	1.12	2.14	0.72	0.59	0.90
Topography						
C50.0 nipple	Ref					
C50.1 central portion of breast	1.01	0.68	1.50	1.14	0.90	1.45
C50.2 upper-inner quadrant of breast	1.06	0.75	1.51	1.16	0.93	1.44
C50.3 lower-inner quadrant of breast	0.95	0.64	1.40	1.23	0.97	1.57
C50.4 upper-outer quadrant of breast	1.01	0.75	1.35	1.12	0.92	1.37
C50.5 lower-outer quadrant of breast	1.02	0.69	1.52	1.02	0.82	1.29
C50.6 axillary tail of breast	0.39	0.18	0.82	1.11	0.77	1.57
C50.8 overl. lesion of breast	0.83	0.62	1.11	1.13	0.92	1.38
C50.9 breast, NOS	0.74	0.56	0.97	1.16	0.96	1.42
Grade						
B-Cell	Ref					
Grade I (Well diff)	7.27	3.38	15.64	1.02	0.59	1.87
Grade II (Mod diff)	8.09	3.97	16.51	1.02	0.59	1.86
Grade III (Poor diff)	6.03	2.97	12.26	1.05	0.61	1.92
Grade IV (Undiff anaplastic)	5.55	2.52	12.19	0.92	0.52	1.74
Laterality						
Bilateral invasive	Ref					
Left	0.95	0.63	1.43	1.06	0.80	1.43
Paired site, late	0.81	0.37	1.80	2.65	1.20	5.35
Right	0.96	0.64	1.45	1.03	0.78	1.39
Extent						
Distant metastasis	Ref					
Localised	1.73	1.46	2.07	0.90	0.82	0.99
Regional	1.61	1.43	1.80	0.88	0.80	0.96
Theta (*θ*)	0.0013			5.2		

**Table 5:** Weibull with gamma frailty (Model 3).

Variables	Weibull with gamma frailty					
	Frequentist			Bayesian approach		
	HR	2.5%	97.5%	HR	2.5%	97.5%
Sex						
Female	Ref					
Male	0.96	0.64	1.44	—	—	—
Age	0.99	0.99	1.00	1.00	1.00	1.01
Marital						
Divorce	Ref					
Married	1.37	1.03	1.81	0.79	0.65	0.96
Single	1.09	0.75	1.58	0.78	0.63	0.98
Widowed	1.57	1.14	2.18	0.72	0.58	0.89
Topography						
C50.0 nipple	Ref					
C50.1 central portion of breast	1.00	0.67	1.50	1.17	0.92	1.49
C50.2 upper-inner quadrant of breast	1.04	0.73	1.49	1.18	0.95	1.49
C50.3 lower-inner quadrant of breast	0.95	0.64	1.40	1.26	0.99	1.63
C50.4 upper-outer quadrant of breast	1.01	0.75	1.35	1.14	0.93	1.41
C50.5 lower-outer quadrant of breast	1.01	0.68	1.50	1.05	0.83	1.33
C50.6 axillary tail of breast	0.38	0.18	0.80	1.25	0.88	1.81
C50.8 overl. lesion of breast	0.82	0.61	1.10	1.15	0.94	1.43
C50.9 breast, NOS	0.73	0.55	0.97	1.19	0.97	1.47
Grade						
B-Cell	Ref					
Grade I (Well diff)	7.13	3.33	15.26	1.08	0.55	2.38
Grade II (Mod diff)	7.90	3.88	16.09	1.08	0.56	2.37
Grade III (Poor diff)	5.91	2.91	11.98	1.12	0.58	2.46
Grade IV (Undiff anaplastic)	5.44	2.49	11.91	0.98	0.49	2.18
Laterality						
Bilateral invasive	Ref					
Left	0.92	0.61	1.39	1.10	0.82	1.51
Paired site, late	0.78	0.36	1.73	2.51	1.05	5.06
Right	0.94	0.62	1.42	1.08	0.80	1.47
Extent						
Distant metastasis	Ref					
Localised	1.73	1.45	2.07	0.91	0.83	0.99
Regional	1.60	1.43	1.79	0.88	0.81	0.96
Theta(*θ*)	0.98			<0.001		

## Data Availability

Data were obtained from the Cancer Registry in King Faisal Specialist Hospital (KFSH) and the Research Centre (RC) upon request. To have access to the data, a formal application of request must be submitted to info@shc.gov.sa or through the cancer registry website: https://nhic.gov.sa/eServices/Pages/manageregisters.aspx.

## References

[1] Tarone R. E. (2006). Breast cancer trends among young women in the United States. *Epidemiology*.

[2] Al-Qahtani M. S. (2007). Gut metastasis from breast carcinoma. *Saudi Medical Journal*.

[3] Lozano R., Naghavi M., Foreman K. (2012). Global and regional mortality from 235 causes of death for 20 age groups in 1990 and 2010: a systematic analysis for the global burden of disease study 2010. *The Lancet*.

[4] Mokdad A. H., Jaber S., Aziz M. I. A. (2014). The state of health in the Arab world, 1990–2010: an analysis of the burden of diseases, injuries, and risk factors. *The Lancet*.

[5] Ibrahim E. M., Zeeneldin A. A., Sadiq B. B. (2008). The present and the future of breast cancer burden in the Kingdom of Saudi Arabia. *Medical Oncology*.

[6] Elkum N., Dermime S., Ajarim D. (2007). Being 40 or younger is an independent risk factor for relapse in operable breast cancer patients: the Saudi Arabia experience. *BMC Cancer*.

[7] Zabicki K., Colbert J. A., Dominguez F. J. (2006). Breast cancer diagnosis in women ≤40 versus 50 to 60 years: increasing size and stage disparity compared with older women over time. *Annals of Surgical Oncology*.

[8] Anders C. K., Hsu D. S., Broadwater G. (2008). Young age at diagnosis correlates with worse prognosis and defines a subset of breast cancers with shared patterns of gene expression. *Journal of Clinical Oncology*.

[9] Amran S. E., Abdullah M. A. A., Kek S. L., Jamil S. A. M. (2017). Analysis of survival in breast cancer patients by using different parametric models. *Journal of Physics: Conference Series*.

[10] Weibull W., Rockey K. C. (1962). Fatigue testing and analysis of results. *Journal of Applied Mechanics*.

[11] Omariba D. W. R., Beaujot R., Rajulton F. (2007). Determinants of infant and child mortality in Kenya: an analysis controlling for frailty effects. *Population Research and Policy Review*.

[12] Baghestani A., Moghaddam S., Majd H., Akbari M., Nafissi N., Gohari K. (2015). Survival analysis of patients with breast cancer using weibull parametric model. *Asian Pacific Journal of Cancer Prevention*.

[13] Nasejje J. B., Mwambi H. G., Achia T. N. O. (2015). Understanding the determinants of under-five child mortality in Uganda including the estimation of unobserved household and community effects using both frequentist and Bayesian survival analysis approaches. *BMC Public Health*.

[14] Martino S., Akerkar R., Rue H. (2010). Approximate Bayesian inference for survival models. *Scandinavian Journal of Statistics*.

[15] Al-Amri F. A., Saeedi M. Y., Al-Tahan F. M. (2015). Breast cancer correlates in a cohort of breast screening program participants in Riyadh, KSA. *Journal of the Egyptian National Cancer Institute*.

[16] Almuazzi R. F., Almuhayfir A. A., Ahmed H. G. (2017). Breast cancer in Saudi Arabia and its possible risk factors. *Journal of Cancer Policy*.

[17] Bolstad W. M., Manda S. O. (2001). Investigating child mortality in Malawi using family and community random effects: a Bayesian analysis. *Journal of the American Statistical Association*.

[18] Gohari M. R., Winchester M. R., Mahmoudi M., Mohammed K., Pasha E., Khodabakhshi R. (2006). Recurrence in breast cancer. Analysis with frailty model. *Saudi Medical Journal*.

[19] Kazembe L., Clarke A., Kandala N.-B. (2012). Childhood mortality in sub-Saharan Africa: cross-sectional insight into small-scale geographical inequalities from Census data. *BMJ Open*.

[20] Martinez M. E., Unkart J. T., Tao L. (2017). Prognostic significance of marital status in breast cancer survival: a population-based study. *PLoS One*.

[21] Gutierrez R. G. (2002). Parametric frailty and shared frailty survival models. *The Stata Journal: Promoting Communications on Statistics and Stata*.

[22] Govindarajulu U. S., Lin H., Lunetta K. L., D’Agostino R. B. (2011). Frailty models: applications to biomedical and genetic studies. *Statistics in Medicine*.

[23] Klein J. P. (1992). Semiparametric estimation of random effects using the cox model based on the EM algorithm. *Biometrics*.

[24] Kosorok M. R., Lee B. L., Fine J. P. (2004). Robust inference for univariate proportional hazards frailty regression models. *The Annals of Statistics*.

[25] Nielsen G. G., Gill R. D., Andersen P. K., Sorensen T. I. (1992). A counting process approach to maximum likelihood estimation of frailty models. *Scandinavian Journal of Statistics*.

[26] Sargent D. J. (1998). A general framework for random effects survival analysis in the cox proportional hazards setting. *Biometrics*.

[27] Vaupel J. W., Manton K. G., Stallard E. (1979). The impact of heterogeneity in individual frailty on the dynamics of mortality. *Demography*.

[28] Austin P. C. (2017). A tutorial on multilevel survival analysis: methods, models and applications. *International Statistical Review*.

[29] Martino S., Rue H. (2009). *Implementing Approximate Bayesian Inference Using Integrated Nested Laplace Approximation: A Manual for the Inla Program*.

[30] Martins T. G., Rue H. (2014). Extending integrated nested laplace approximation to a class of near-Gaussian latent models. *Scandinavian Journal of Statistics*.

[31] Ferkingstad E., Rue H. (2015). Improving the INLA approach for approximate Bayesian inference for latent Gaussian models. *Electronic Journal of Statistics*.

[32] Martins T. G., Simpson D., Lindgren F., Rue H. (2013). Bayesian computing with INLA: new features. *Computational Statistics & Data Analysis*.

[33] Rue H., Held L. (2005). *Gaussian Markov Random Fields: Theory and Applications*.

[34] Rue H., Steinsland I., Erland S. (2004). Approximating hidden Gaussian Markov random fields. *Journal of the Royal Statistical Society: Series B (Statistical Methodology)*.

[35] Rondeau V., Mazroui Y., Gonzalez J. R. (2012). Frailtypack: an R package for the analysis of correlated survival data with frailty models using penalized likelihood estimation or parametrical estimation. *Journal of Statistical Software*.

[36] Michaelson H., Hanson T., Jara A., Zhang J. (2015). Modeling county level breast cancer survival data using a covariate-adjusted frailty proportional hazards model. *The Annals of Applied Statistics*.

[37] Ghadimi M., Mahmoodi M., Mohammad K., Zeraati H., Rasouli M., Sheikhfathollahi M. (2011). Family history of the cancer on the survival of the patients with gastrointestinal cancer in northern Iran, using frailty models. *BMC Gastroenterology*.

[38] Gurmu S. E. (2018). Assessing survival time of women with cervical cancer using various parametric frailty models: a case study at Tikur anbessa specialized hospital, Addis Ababa, Ethiopia. *Annals of Data Science*.

[39] Feng S., Wolfe R. A., Port F. K. (2005). Frailty survival model analysis of the national deceased donor kidney transplant dataset using Poisson variance structures. *Journal of the American Statistical Association*.

[40] Bakhshi E., Sheikhaliyan A., Atashgar K., Kooshesh M., Biglarian A. (2017). Survival analysing of the breast cancer patients using cure model. *Iranian Red Crescent Medical Journal*.

[41] Wienke A., Lichtenstein P., Czene K., Yashin A. I., Auget J.-L., Balakrishnan N., Mesbah M., Molenberghs G. (2007). The role of correlated frailty models in studies of human health, ageing, and longevity BT-advances in statistical methods for the health sciences: applications to cancer and AIDS studies, genome sequence analysis, and survival analysis. *Advances in Statistical Methods for the Health Sciences*.

[42] Gorfine M., Hsu L., Parmigiani G. (2013). Frailty models for familial risk with application to breast cancer. *Journal of the American Statistical Association*.

[43] Giuliano A. E., Connolly J. L., Edge S. B. (2017). Breast cancer-major changes in the American Joint Committee on cancer eighth edition cancer staging manual. *CA: A Cancer Journal for Clinicians*.

[44] Kamińska M., Ciszewski T., Łopacka-Szatan K., Miotła P., Starosławska E. (2015). Breast cancer risk factors. *Menopausal Review*.

[45] García-Closas M., Brinton L. A., Lissowska J. (2006). Breast cancer risk factors by clinically important tumour characteristics. *British Journal of Cancer*.

[46] Grover G., Seth D. (2014). Application of frailty models on advance liver disease using gamma as frailty distribution. *Statistics Research Letters*.

